# Supplementary material for: Mitochondrial gene expression signature predicts prognosis of pediatric acute myeloid leukemia patients
Source: Front Oncol. 2023 Feb 9;13:1109518. doi: 10.3389/fonc.2023.1109518 (PMC9947241; doi:10.3389/fonc.2023.1109518)
Supplement: Supplementary file 1 [file DataSheet_1.pdf]

Supplementary Methods

S1: Risk Assessment by karyotyping and mutation analysis

Conventional cytogenetics were done at baseline to identify translocations, inversions, deletion as well as other chromosomal abnormalities for risk stratification of pediatric AML. Mutation profiling of *RUNX1-RUNX1T1* (Runt-related transcription factor 1-RUNX1 partner transcriptional co-repressor 1 fusion transcript), *CBFB-MYH11* (Core binding factor beta-myosin heavy chain 11 fusion transcript), FLT3-ITD (Fms like tyrosine kinase 3-internal tandem duplication), and *NPM1* (Nucleophosmin 1) by reverse transcriptase polymerase chain reaction (PCR) were performed at baseline for risk assessment as per European LeukemiaNet (ELN) recommendation[1].

S2: Treatment protocol

All patients were treated with uniform induction protocol, i.e., 3+7 regimen including daunorubicin 60 mg/m^2 day 1-3 and cytarabine $100mg/m^2$ continuous infusion day 1-7. Consolidation therapy with three cycles of high dose cytarabine at $18g/m^2$ were given to patients after achieving complete remission (CR) whereas repeated induction with ADE regimen (cytarabine: 100 mg/m2 twice daily, day 1-10; daunorubicin: 50 mg/m2, day 1-3; and etoposide: 100 mg/m2, day 1-5) were used for refractory or relapse cases. Patients at CR2 underwent allogeneic hematopoietic stem cell transplantation with matched sibling donor if available [2,3].

S3: Isolation of bone marrow mononuclear cells (BMMCs), DNA and RNA extraction and quantification

BM samples collected from all the patients and controls were layered onto the histopaque in 15 ml falcon tube followed by centrifugation at 400g for 30 min at room temperature. The mononuclear cells layer was carefully taken out after centrifugation and washed twice with phosphate buffer saline (PBS). BMMC were then stored for DNA isolation using isopropanol precipitation method and total RNA was isolated using TRIzol method as per manufacturer's protocols. Quality of DNA and RNA was checked by Nanodrop 1000 (Thermo Fisher) and integrity of RNA was checked by TapeStation (Agilent)

S4: Estimation of mitochondrial DNA copy number

Relative mtDNA copy number was assessed by fluorescent DNA binding dye SYBR based quantitative real time PCR using, with Roche Light Cycler 480 II. The relative mitochondrial DNA copy number was normalized to expression of nuclear gene *ACTB*, which was chosen as a nuclear housekeeping gene. The mitochondrial DNA copy number, normalized to copies per cell, in each subject and control sample was calculated using the following formula: $2^\wedge[\mathrm{Ct}(\beta{-}\mathrm{actin}) - \mathrm{Ct}\ (\text{minor arc})]$ (Ct being respective cycle thresholds). Relative mitochondrial DNA copy numbers of patients were then compared with controls

S5: Library Construction and sequencing

The sequencing library was prepared by random fragmentation of the cDNA sample, followed by 5' and 3' adapter ligation, after end-repair and the addition of an 'A' base and SPRI clean-up. The prepared cDNA library was amplified using PCR for the enrichment of the adapter-ligated fragments. The individual libraries were quantified using a NanoDrop spectrophotometer (Thermo Scientific) and validated for quality with a Bioanalyzer (Agilent Technologies). Adapter-ligated fragments were then PCR amplified and gel purified. cDNA library was used for sequencing which was carried out on Illumina Hiseq 4000 NGS platform.

S6: Processing of the reads and differential expression analysis

The quality of raw reads was first checked by FastQC version v0.11.8. Trimmomatic was performed to remove adapter sequences and low-quality reads for further analysis. The trimmed reads were then aligned to reference Human genome (hg38) using HISAT2 tool. SAM tool was used to convert SAM files into BAM files which was used for quantification and estimation of aligned reads by StringTie (v2.0.6). Lastly differential expression analysis was performed by limma Bioconductor package (version 3.48.1). Absolute fold change value ≥ 2 (a $\geq$ two-fold change in expression, either upregulated or downregulated) and adjusted p value ($q \leq 0.05$) threshold was used for the identification of differentially expressed genes (DEGs). Volcano plots were made with log fold change; p value and adjusted p value of the transcripts were calculated using devtools in R package (version 3.6.1). Gene name of the corresponding transcripts with significant p value, adjusted p value and log 2-fold

difference compared to controls, were fetched using gProfilerR library in R package. Exclusive differentially expressed genes were identified among the groups by making Venn diagram using Venny (Venny 2.1) online tool.

S7: Construction of protein-protein interaction network, determination of HUB genes and MCODE analysis

A interactive network of proteins encoded DEGs present in the respective groups were constructed using Search Tool for the Retrieval of Interacting Genes/Proteins (STRING) database [4] in Cytoscape. Cytohubba plugin was used for identifying Hub genes among the whole interactome network. Those genes which got enriched using at least six different topological algorithms among Degree, Edge Percolated Component (EPC), Maximum Neighbourhood Component (MNC), Density of Maximum Neighbourhood Component (DMNC), Maximal Clique Centrality (MCC) and centralities based on shortest paths, such as Bottleneck (BN), Eccentricity, Closeness, Radiality, Betweenness, and Stress were considered as Hub genes[5]. Cytoscape plug-in MCODE clustering algorithm was used for identifying the maximum ranked cluster which has a highly interconnected region , also known as seed nodes as well as their neighbour nodes in the whole network [6]. Subcellular location of the genes was identified from the gene ontology table. Compartment mitochondria score was used for selecting mitochondrial related genes among all the AML subgroups.

References

1. Döhner H, Estey E, Grimwade D, Amadori S, Appelbaum FR, Büchner T, et al. Diagnosis and management of AML in adults: 2017 ELN recommendations from an international expert panel [Internet]. Vol. 129, Blood. American Society of Hematology; 2017 [cited 2021 Apr 10]. p. 424–47. Available from: http://ashpublications.org/blood/article-pdf/129/4/424/1402046/blood733196.pdf

2. Tyagi A, Pramanik R, Chaudhary S, Chopra A, Bakhshi S. Cytogenetic Profiles of 472 Indian Children with Acute Myeloid Leukemia. Indian Pediatr. 2018;55(6):469–73.

3. Arora S, Pushpam D, Tiwari A, Choudhary P, Chopra A, Gupta R, et al. Allogeneic

hematopoietic stem cell transplant in pediatric acute myeloid leukemia: Lessons learnt from a tertiary care center in India. Pediatr Transplant [Internet]. 2020 [cited 2021 Apr 12]; Available from: https://pubmed.ncbi.nlm.nih.gov/33142026/

4. Franceschini A, Szklarczyk D, Frankild S, Kuhn M, Simonovic M, Roth A, et al. STRING v9.1: protein-protein interaction networks, with increased coverage and integration. Nucleic Acids Res. 2012 Nov;41(D1):D808–15.

5. Chin CH, Chen SH, Wu HH, Ho CW, Ko MT, Lin CY. cytoHubba: Identifying hub objects and sub-networks from complex interactome. BMC Syst Biol. 2014;8(4):S11.

6. Bader GD, Hogue CWV. An automated method for finding molecular complexes in large protein interaction networks. BMC Bioinformatics. 2003 Jan;4:2.
