# Supplementary material for: Mitochondrial gene expression signature predicts prognosis of pediatric acute myeloid leukemia patients
Source: Front Oncol. 2023 Feb 9;13:1109518. doi: 10.3389/fonc.2023.1109518 (PMC9947241; doi:10.3389/fonc.2023.1109518)
Supplement: Supplementary file 2 [file DataSheet_2.pdf]

Supplementary Figures and Tables

Supplementary Figure 1: RNA sequencing data analysis of pediatric AML patient samples and identification of differentially regulated genes for validation

Supplementary Figure 2: Association of mtDNA copy number with survival outcome Kaplan Meier curves representing association of mtDNA copy number with (A) event free survival and (B) overall survival of pediatric AML patients; Subgroup analysis of acute myeloid leukemia showing exclusive deregulation of validated genes among AMLCN_L and AMLCN_H group. The expression of *SLC25A3*(C), *SDHC*(D), *RACK1*(E) and *FASTKD1*(F) were significantly higher in AMLCN_H group as compared to AMLCN_L.

Supplementary Figure 3: Forest plot showing the impact of mitochondrial prognostic gene signature risk score category on survival outcome in various clinically relevant subgroups of pediatric Acute myeloid leukemia

Supplementary Table 1: Primer sequence of all the selected differentially expressed genes (DEGs) for validation by q-RT PCR

Supplementary Table 2: Baseline characteristic features of pediatric AML patients (n=143)

Supplementary Table 3: Baseline characteristic features of external validation cohort of TCGA adult AML patients (n=179)

Supplementary Table 4: List of overlapping Hub genes in three subgroups of Acute myeloid leukemia

Supplementary Table 5: MCODE (molecular complex detection) analysis of all the three groups for identification of seed genes

Supplementary Table 6: List of genes selected for validation among all the groups based on mitochondrial compartment score, *Cytohubba* and *MCODE*

Supplementary Table 7: List of genes showing significant correlation with mitochondrial DNA copy number using Pearson's correlation

Supplementary Table 8: List of significant genes out of all the validated differentially expressed genes (DEGs) using Bootstrapping method (10000 resample)

Supplementary Table 9: Univariable and multivariable analysis of clinical features with overall survival

Supplementary Table 10: Multivariable cox regression analysis of mtDNA copy number, ELN risk group and gene signature risk score on overall survival and event free survival of pediatric AML patients

Supplementary Table 11: Association of Risk score with clinical features in TCGA dataset (n=179)

Supplementary Table 12: Comparison of our gene signatures with gene signature of several studies on pediatric AML

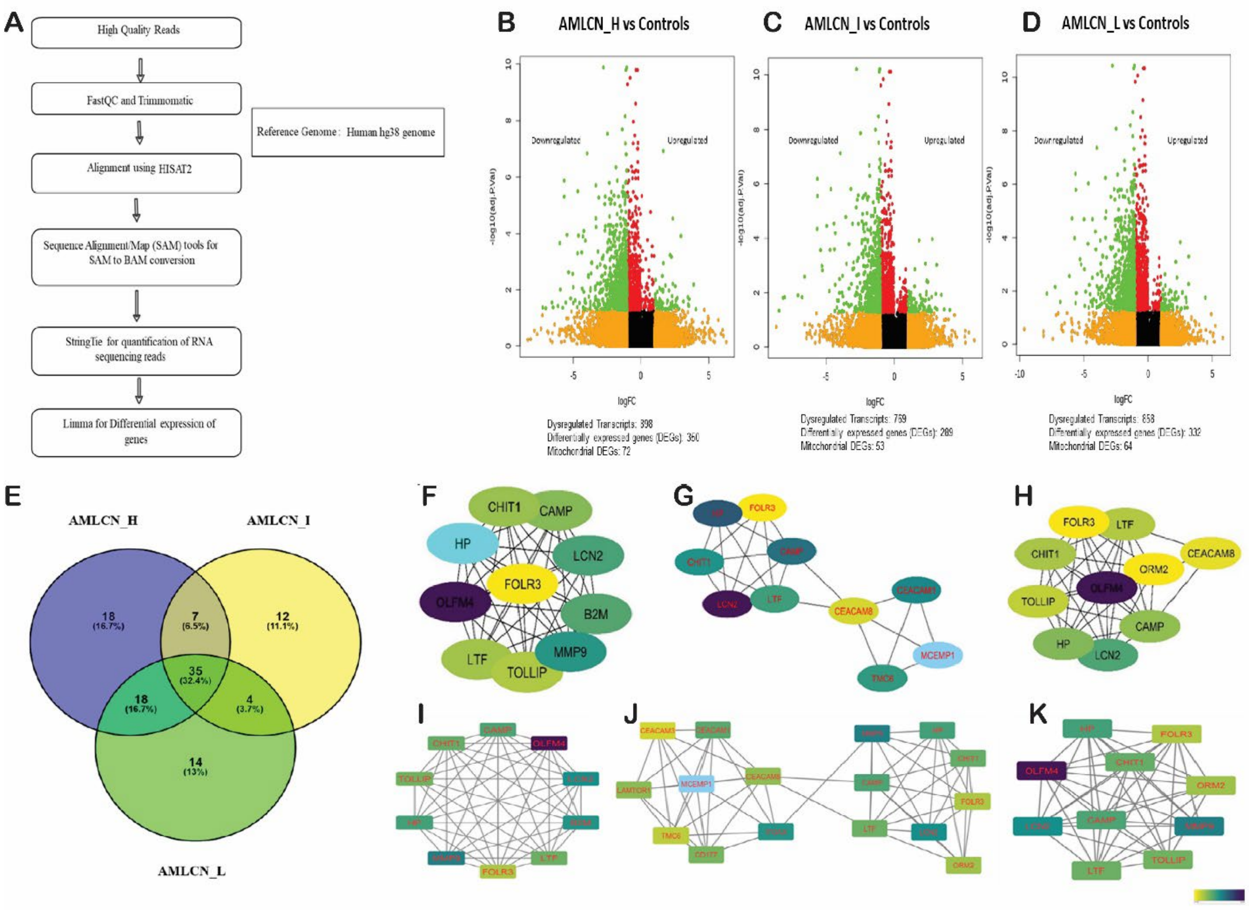

Figure S1: **RNA sequencing data analysis:** (A)Workflow of RNA sequencing data analysis using various bioinformatic tools; (B)-(D) Volcano plots representing all dysregulated transcripts among AMLCN_H (B), AMLCN_I (C) and AMLCN_L (D) groups compared to controls. Green dots represent significantly differentially expressed transcripts in the three subgroups of AML compared to controls; High AMLCN_H (more than 75th percentile); intermediate AMLCN_I (50th to 75th percentile) and low AMLCN_L (lower than 50th percentile) relative mitochondrial DNA copy number (E) Venn diagram representing mitochondrial associated differentially expressed genes (DEGs) among three groups of AML (AMLCN_H, AMLCN_I, AMLCN_L)*; (F)-(H) Hub gene based on Maximal Clique Centrality (MCC) (F): AMLCN_H; (G) AMLCN_I; (H) AMLCN_L; (I)-(K): MCODE (molecular complex detection) analysis showing most interactive clusters among A: (I): AMLCN_H; (J) AMLCN_I; (K) AMLCN_L. *High AMLCN_H (more than 75th percentile); intermediate AMLCN_I (50th to 75th percentile) and low AMLCN_L (lower than 50th percentile) relative mitochondrial DNA copy number

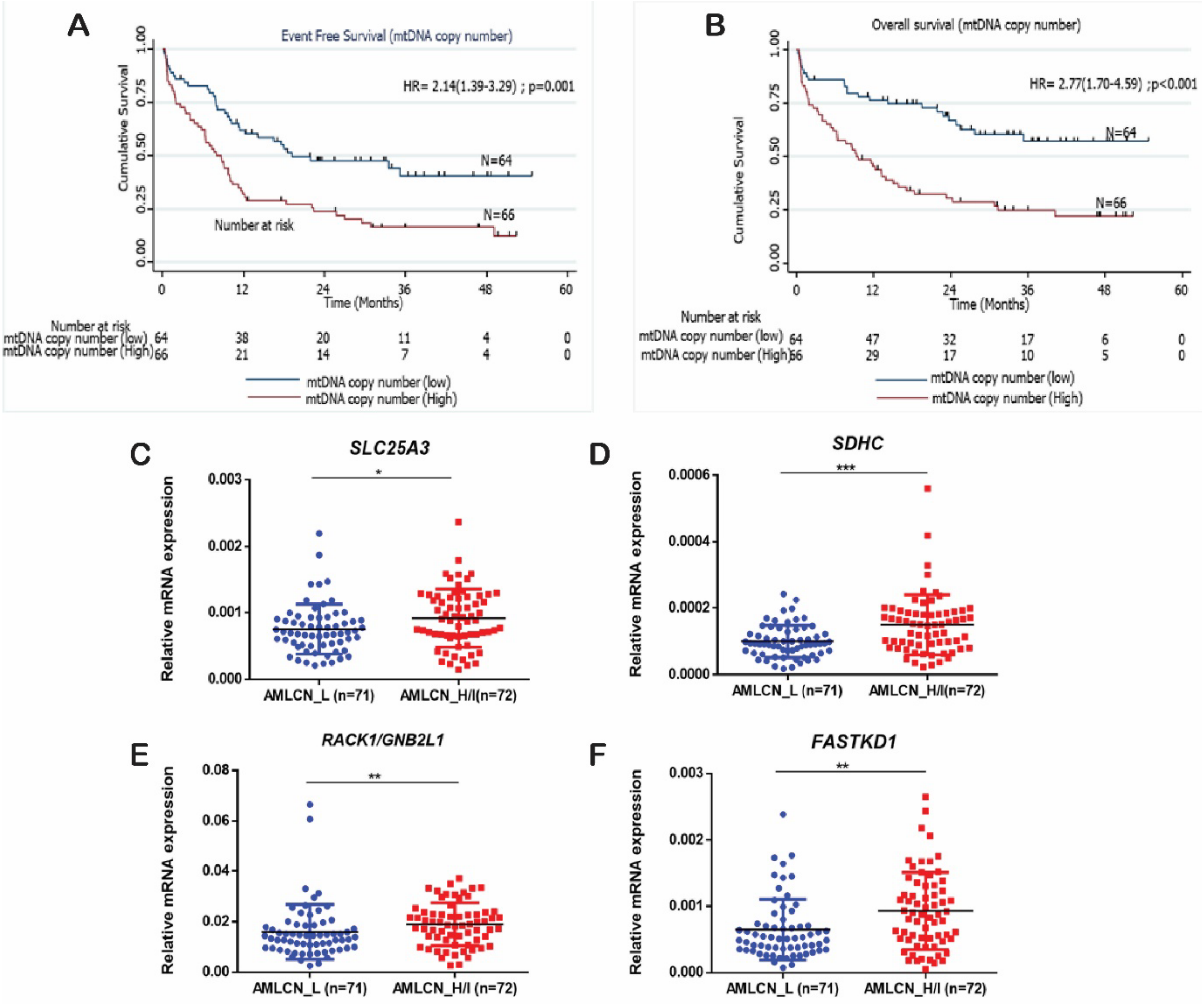

Figure S2: Association of mtDNA copy number with survival outcome Kaplan Meier curves representing association of mtDNA copy number with (A) event free survival and (B) overall survival of pediatric AML patients; Subgroup analysis of acute myeloid leukemia showing exclusive deregulation of validated genes among AMLCN_L and AMLCN_H group. The expression of *SLC25A3*(C), *SDHC*(D), *RACK1*(E) and *FASTKD1*(F) were significantly higher in AMLCN_H group as compared to AMLCN_L.

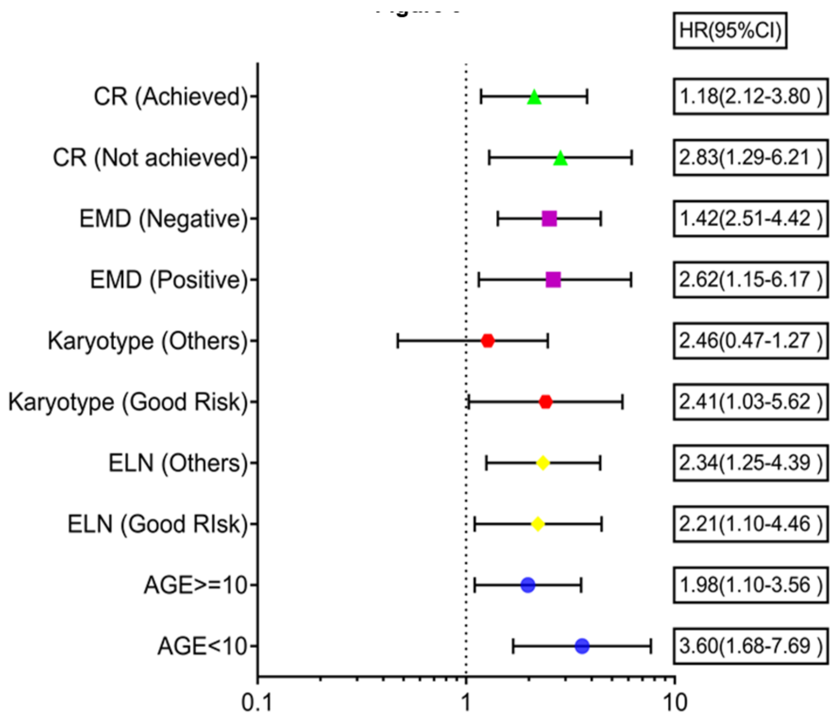

Figure S3: Forest plot showing the impact of mitochondrial prognostic gene signature risk score category on survival outcome in various clinically relevant subgroups of pediatric Acute myeloid leukemia

Table S1: Primer sequence of all the selected differentially expressed genes (DEGs) and 18S for validation by qRT PCR

S no.	Gene name	Forward (5' to 3')	Reverse (5' to 3')
1	***SLC25A3***	ATGGAAGGGACTGTTTGCCC	AGAGACTCTGGCATCTCGGG
2	***LONP1***	GATGCCAAGGGTGACAAGGA	TCACCAGGTAGTCATTGGCG
3	***SDHC***	AAAGCTGTGGGTCGAAGAGG	GTTAAAACAGCGGGTCCCCA
4	***GNB2L1/RACK1***	CAGGAGAGGTTGTGGTGCTA	GTGCCACGAAGGGTCATCTG
5	***FASTKD1***	AAAAGCAGAAGACCAGCCTGT	ACACATTCACCAGGGTATCGTC
6	***MRPL51***	CTGATGACCTGCACAACCTT	GCCTTTTCCGAAGTTCTCAGC
7	***ATP5J***	CCCTTGTGGGGGAAAAGGC	CGGAGTGGGTGAGTGCG
8	***FASLG***	ACCTTGAGACCACAGGGTTC	CTCCCTCTTCACATGGCAGC
9	***CLIC1***	CTAGGGGAGAGAGTCCCTGA	CAATTCGACCTGCGGTTGTTC
10	***HRK***	ATGGCATTGAGAGGCTTGCT	TCCGAGGACACTTTTGTGGG
11	***ALAS2***	TCCTGTTTGGTATTGGACGCT	CCTCCAGCCTTTGTTGCCTTA
12	***SLC25A21***	AACCCTCGCTTTCCAGCAGT	GTGGGCTGAGATGCGTCAA
13	***CYP1B1***	GCTCACCTCTCGTTCGCTC	TCGCCATTCAGCACCACTAT
14	***MMP9***	GACCAAGTGGGCTACGTGAC	TGTATCCGGCAAACTGGCTC
15	***SNCA***	ATCGTTGGAACTACCAGAGTCAC	TCTTCCCTGAAAGAGAAAAACCT
16	***DHFR***	ACATTGTCGGGCTTTTCACAT	CAAACAAGGTGGCAACATTTATC
17	***GLUD1***	AGTCCAAAGAATGCAGTCACTTTT	CAGCCAGACTCCAAACAGGG
18	***SLC25A29***	CAGACACTCCAGCCCCTTTC	AGTTAGAGGTCCCTGGGTGG
19	***LIG1***	TCAGAACCAACAAGGCGAGG	CGTCCAACTCATGCCCTGTA
20	***OLFM4***	AAGGACTGTATTGGGTGGCG	GCCATAGGTGATCCGCAACT
21	***18S***	GTAACCCGTTGAACCCCATT	CCATCCAATCGGTAGTAGCG

Table S2: Baseline characteristic features of pediatric AML patients (n=143)

**Characteristics**	**Number (%)**
**Median age (years)**	10 (0.8-18)
**Sex**	
Male	87 (60.8)
Female	56 (39.2)
**Haematological parameters**	
Median haemoglobin, (g/dL)	7.4 (2.1-14.6)
Median total leucocyte count, ($\times10^3/\mu L$)	24.38 (0.76-314.3)
Hyperleukocytosis, ($>50\times10^3/\mu L$)	45 (31.46)
Median platelet count, ($\times10^3/\mu L$)	35.00(4-276)
**Clinical features at presentation**	
Fever	112(78.3)
Chloroma	27 (18.9)
**Cytogenetics (n=130)**	
Normal	36 (27.5)
t(8;21) & inv (16)*	52 (39.7)
Complex karyotype	8 (6.1)
Other	19 (14.5)
Failed cytogenetics	16(12.2)
**Molecular analysis (n= 122)**	
FLT3 ITD	17 (13.8)
RUNX1-RUNX1T1	53 (43.1)
CBFB-MYH11	6 (4.9)
NPM1	5 (4.1)
Negative	41 (33.6)
**ELN risk stratification** (n=134)**	
Good	67 (50)
Intermediate	37 (27.6)
Poor	30 (22.4)
**Complete remission (n=143)**	
Achieved	104 (72.7%)
Not achieved	39 (27.3%)

* t(8;21)= t(8;21)(q22;q22) *RUNX1-RUNX1T1*; inv(16)= inv(16)(p13.1;q22) *CBFB-MYH11*

**ELN risk stratification was done using both cytogenetics and molecular markers in 134 patients. However, for 12 patients risk stratification was done with only cytogenetics and in 20 patients, it was done by only molecular analysis.

Median values were reported with range

n: number of patients; AML: Acute Myeloid Leukemia; Normal cytogenetics: 46,XX/46,XY; Failed cytogenetics: metaphases could not be isolated; *FLT3 ITD*: FMS-like tyrosine kinase internal tandem duplication; *RUNX1-RUNX1T1*: runt-related transcription factor 1-RUNX1 partner transcriptional co-repressor 1 fusion transcript; *CBFB-MYH11*: core binding factor beta-myosin heavy chain 11 fusion transcript; *NPM1*: Nucleophosmin 1; ELN: European LeukemiaNet.

Table S3: Baseline characteristic features of external validation cohort of TCGA adult AML patients (n=179)

Characteristics	Number (%)
**Median age (years)**	58 (18-88)
**Sex**	
Male	95 (53.1)
Female	84 (46.9)
**Race**	
Asian	2 (1.1)
Black or African American	13(7.3)
White	162(90.5)
Not available	2(1.1)
**Cytogenetics**	
Normal	89 (49.7)
t(8;21) & inv (16)	15 (8.4)
Complex karyotype	24(13.4)
Other	33 (17.4)
Failed cytogenetics	18(10.1)

* t(8;21)= t(8;21)(q22;q22) *RUNX1-RUNX1T1*; inv(16)= inv(16)(p13.1;q22); TCGA: The cancer genome atlas; AML: Acute myeloid leukemia

Table S4: List of overlapping Hub genes in three subgroups of Acute myeloid leukemia

#Protein ID	Gene name	*Expression in AMLCN_H	*Expression in AMLCN_I	*Expression in AMLCN_L
ENSP00000348170	HP	-3.1902	-3.17448	-3.05683
ENSP00000354003	GYPA	-7.06792	-4.26827	-6.09589
ENSP00000361405	MMP9	-69.1165	-110.06	-50.0099
ENSP00000454623	ITGAX	-2.14021	-2.11234	-2.14021
ENSP00000263341	IL1B	-2.68106	-3.01207	-2.54287
ENSP00000332369	ALAS2	-10.3003	-5.26182	-9.62213
ENSP00000376544	CD79B	-2.79991	-9.86539	-8.91179
ENSP00000085219	CD22	-3.81718	-3.89379	-3.85420

#Protein IDs of the corresponding hub genes obtained from the network analysis

*Log2fold change expression of hub genes with respect to the control samples in our RNA sequencing samples with high AMLCN_H (more than 75th percentile) intermediate AMLCN_I (50th to 75th percentile) and low AMLCN_L (lower than 50th percentile) relative mitochondrial DNA copy number.

Table S5: MCODE (molecular complex detection) analysis of all the three AML subgroups for identification of seed genes

**AMLCN_H**	**Enrichment Network**	**MCODE SOCRE**	**AMLCN_I**	**Enrichment Network**	**MCODE SOCRE**	**AMLCN_L**	**Enrichment Network**	**MCODE SOCRE**
B2M	Clustered	7	*CEACAM8*	Clustered	6	*MMP9*	Clustered	7
FOLR3	Clustered	7	*CD177*	Clustered	6	*OLFM4*	Clustered	8
CAMP	Clustered	7	*ORM2*	Clustered	6	*HP*	Clustered	8
HP	Clustered	7	*LCN2*	Clustered	5	*CAMP*	Clustered	8
LCN2	Clustered	7	*CAMP*	Clustered	5	*LTF*	Clustered	8
LTF	Clustered	7	*MCEMP1*	Clustered	6	*CHIT1*	Clustered	8
CHIT1	Clustered	7	*ITGAX*	Clustered	5	*LCN2*	Clustered	8
OLFM4	Clustered	7	*CHIT1*	Clustered	5	***FOLR3***	**Seed**	**8**
MMP9	**Seed**	**8**	*TMC6*	Clustered	6	*TOLLIP*	Clustered	8
TOLLIP	Clustered	8	*LAMTOR1*	Clustered	6	*ORM2*	Clustered	8
			CEACAM3	Clustered	6			
			MMP9	**Seed**	**6**			
			CEACAM1	Clustered	6			
			HP	Clustered	5			
			LTF	Clustered	5			

			FOLR3	Clustered	5			

MCODE score: Molecular complex detection score; AML: Acute myeloid leukemia; High AMLCN_H (more than 75th percentile); intermediate AMLCN_I (50th to 75th percentile) and low AMLCN_L (lower than 50th percentile) relative mitochondrial DNA copy number

Table S6: List of genes selected for validation among all the groups based on mitochondrial compartment score, *Cytohubba* and *MCODE*

S no.	Gene name	Differentially expressed transcript ID	Compartment mitochondria score (0-5)	Groupwise expression	Expression
1	*SLC25A3*	ENST00000401722	5	AMLCN_H	Upregulation
2	*LONP1*	ENST00000589473	5	AMLCN_H	Downregulation
3	*SDHC*	ENST00000367975	5	AMLCN_H	Upregulation
4	*GNB2L1/RACK1*	ENST00000376817	4.2	AMLCN_H	Upregulation
5	*FASTKD1*	ENST00000453929	5	AMLCN_I	Upregulation
6	*MRPL51*	ENST00000538814	5	AMLCN_I	Downregulation
7	*ATP5J*	ENST00000486002	5	AMLCN_I	Upregulation
8	*FASLG*	ENST00000367721	2.64	AMLCN_L	Downregulation
9	*CLIC1*	ENST00000375784	3.12	AMLCN_L	Upregulation
10	*HRK*	ENST00000257572	5	Common in all AML samples	Downregulation
11	*ALAS2*	ENST00000463868	5	Common in all AML samples	Downregulation
12	*SLC25A21*	ENST00000331299	5	Common in all AML samples	Downregulation
13	*CYP1B1*	ENST00000610745	4.35	Common in all AML samples	Downregulation
14	*GLUD1*	ENST00000277865	5	Common in AMLCN_H & AMLCN_I	Upregulation
15	*SLC25A29*	ENST00000556505	5	Common in AMLCN_H & AMLCN_I	Upregulation
16	*LIG1*	ENST00000597146	3.6	Common in AMLCN_H & AMLCN_I	Downregulation
17	*SNCA*	ENST00000420646	4.6	Common in AMLCN_L & AMLCN_H	Downregulation
18	*DHFR*	ENST00000513048	4.7	Common in AMLCN_L & AMLCN_H	Downregulation
19	*MMP9*	ENST00000372330	2.8	Hub gene and Seed gene in MCODE cluster of AMLCN_H & AMLCN_I	Downregulation
20	*OLFM4*	ENST00000219022	5	MCODE cluster gene	Downregulation

All the genes selected for validation are listed in the table. Selection of genes were based on their mitochondrial compartment score as determined by *Cytoscape*. *SLC25A3, LONP1, SDHC, GNB2L1/RACK1* were selected exclusively from AMLCN_H subgroup. Three genes (*FASTKD1, MRPL51 AND ATP6J*) belongs to AMLCN_I subgroup and two genes *(FASLG & CLIC1*) were selected from AMLCN_L subgroup. Furthermore, three genes (*SLC25A29, GLUD1* and *LIG1*) which were common among AMLCN_H and AMLCN_I and two genes, *DHFR*

and *SNCA* were selected from the common genes among AMLCN_H and AMLCN_L cohort. *MMP9* and *OLFM4* were selected based on the Hub gene and MCODE analysis.

Table S7: List of genes showing significant correlation with mitochondrial DNA copy number using Pearson's correlation

**Gene name**	**Mitochondrial DNA Copy number (n=131)**	
	Correlation coefficient (r)	p value
ATP5J	0.133	0.024
FASTKD1	0.158	0.008
CLIC1	0.165	0.005
RACK1/GNB2L1	0.174	0.003
SDHC	0.172	0.004
SLC25A3	0.155	0.009

n= number of patients

Table S8: Internal validation of all the validated Differentially expressed genes(DEGs) using Bootstrapping method (10000 resample)

**Bootstrap for Variables in the Equation**						
**Genes**	**Beta**	**Bootstrap**				
		**Bias**	**Std. Error**	**Significance**	**95% Confidence Interval**	
					**Lower**	**Upper**
SDHC	0.237	0.006	0.058	<0.001	0.137	0.377
CLIC1	0.179	0.013	0.087	0.022	0.045	0.397
SLC25A29	-0.131	-0.006	0.032	<0.001	-0.207	-0.082

Table S9: Univariable and multivariable analysis of clinical features with overall survival

Variables (n=143)	Categories (n)	Univariable analysis			Multivariable analysis	
		**Median**	**Hazard (95% CI)**	**p value**	**Hazard (95% CI)**	**p value**
**Age(years)**	≤10 (64)	20.63	0.98(0.63-1.52)	0.93	-	-
	>10(79)	21.93				
**Gender**	Male (87)	15.07	0.72(0.45-1.143)	0.16	-	-
	Female (56)	40.23				
**Total leukocyte count (/mm^3)**	<50000(98)	23.77	0.8(0.517-1.238)	0.32	-	-
	>50000(45)	13.88				
**Platelets (/µL)**	≤ 50000(92)	19.53	0.94(0.59-1.48)	0.789	-	-
	>50000(51)	23.33				
**Haemoglobin(g/dl)**	≤8(95)	13.87	0.73(0.47-1.12)	0.16	-	-
	>8(48)	24.87				
**Fever**	Negative (26)	Not reached	1.52(0.82-2.83)	0.18	-	-
	Positive (112)	19.53				
**Chloroma (n=132)**	Negative (105)	20.63	0.56(0.29-1.07)	0.083	1.45(0.74-2.84)	0.27
	Positive (27)	Not reached				
**ELN Risk group (n=134)**	Good (67)	40.23	1.95(1.23-3.09)	0.004	0.59(0.36-0.98)	0.041
	Others (67)	12.27				

CI: Confidence interval; ELN: European LeukemiaNet.

Table S10: Multivariable cox regression analysis of mtDNA copy number, ELN risk group and gene signature risk score on overall survival and event free survival of pediatric AML patients

	**Overall survival**		**Event Free Survival**	
	**Hazard Ratio (95% CI)**	**P value**	**Hazard Ratio (95% CI)**	**P value**
**MtDNA copy number**	1.00(1.00-1.00)	0.012	1.00(1.00-1.00)	0.030
**ELN Risk group**	1.89(1.15-3.13)	0.012	1.60(1.033-2.46)	0.035
**Risk Score**	1.01(1.006-1.013)	<0.001	1.008(1.004-1.011)	<0.001

CI: Confidence interval; MtDNA Mitochondrial DNA; ELN European LeukemiaNet

Table S11: Association of Risk score with clinical features in TCGA dataset (n=179)

Characteristics (n) (%)	Risk score Low	Risk score High	χ^2	P value
**Age (Median years)**				
<57 years (46.9)	48	36	3.488	0.073
≥57 years (53.1)	41	54		
**Sex**				
Male (53.1)	53	42	2.982	0.084
Female (46.9)	36	48		
**Cytogenetics**				
Good Risk (10.5)	12	3	7.074	0.008
Others (89.5)	56	72		

TCGA: The Cancer Genome Atlas.

Table S12: Comparison of our gene signatures with gene signature of several studies on pediatric AML

**Author (Year)**	**Population (Sample size)**	**Conclusion**	**Impact**	**Predictive power of the model (c-index / AUC)**
**Cai et al. (2020) (Ref 33)**	Pediatric AML patients TARGET dataset (n=187)	Three genes such as EEF1A1, RPLP2, RPL19 were reported as a potential prognostic marker for pediatric AML.	OS	AUC:0.669
**Duployez et al. (2019)**<br>**(Ref 34)**	Pediatric AML patients (n==228)	They developed leukemic stem cell gene expression (LSC17) score and demonstrated the prognostic relevance of LSC17 score. This LSC17 score was also found as an independent prognostic factor in multivariable analysis.	OS and EFS	Not mentioned
**Nguyen et al. (2019)**<br>**(Ref 35)**	Pediatric AML patients (n=228) and TARGET dataset (n=257)	Four gene signatures including SOCS2, IL2RA, NPDC1, PHGDH were reported as an independent prognostic parameter in multiple cohorts of patient with AML. The 4 gene signature also improved prognostication based on ELN classification.	EFS and OS	Not Mentioned
**Shiba et al. (2019)**<br>**(Ref 36)**	Pediatric AML patients (n= 139)	They performed RNA seq analysis and identifies five novel gene rearrangements and reported disease causing gene alterations in pediatric AML patients	OS	Not Mentioned
**Balgobind et al. (2011)**<br>**(Ref 37)**	Pediatric AML patients (n=237)	Authors have identified gene signature of 75 probes sets for predicting most important non-random cytogenetic aberrations with 99% accuracy.	Cytogenetics aberration	Predictive percentage 92% and 99% in discovery and independent validation cohort
**Jiang et. al. (2012)**<br>**(Ref 38)**	Pediatric AML patients from TARGET dataset (n=187) and GSE37642 dataset for validation (n=417)	They have reported gene signature of immune checkpoint– associated genes that includes three genes i.e., STAT1, BATF and EML4. This gene signature was found independently predictive of OS in discovery and validation cohort of pediatric AML patients.	OS	AUC values were 0.654, 0.711, and 0.681, respectively, in TARGET dataset for 1-, 3-, and 5-year survival rates<br>Validation cohort AUC values were 1, 3, and 5 years, respectively, of

				0.569, 0.587, and 0.571
**Our Paper**	Pediatric AML (n=143) and TCGA adult AML patients (n=179)	We identified novel mitochondria-related 3 genes-based signature which was found an independent prognostic factor for OS and EFS in pediatric AML cohort. The 3 gene signature was validated in an external cohort of AML patients.<br><br>These 3 gene-based signatures were also predictive of survival over and above ELN risk classification.	OS and EFS	Discovery cohort AUC 0.747<br><br>Validation cohort AUC 0.649

AML: Acute myeloid leukemia; OS: overall survival; EFS; Event free survival; AUC; Area Under Curve; ELN: European LeukemiaNet
